# Up-regulation of RIP1 and IPS-1 in chronic HBV infected patients

**DOI:** 10.1590/1678-4685-GMB-2018-0071

**Published:** 2019-08-19

**Authors:** Minoo Safari-Arababadi, Mohammad Hossein Modarressi, Mohammad Kazemi Arababadi

**Affiliations:** 1 Department of Genetics, Faculty of Basic Sciences, Science and Research Branch, Islamic Azad University, Tehran, Iran; 2 Department of Medical Genetics, School of Medicine, Tehran University of Medical Sciences, Tehran, Iran; 3 Immunology of Infectious Diseases Research Center, Research Institute of Basic Medical Sciences, Rafsanjan University of Medical Sciences, Rafsanjan, Iran; 4 Department of Laboratory Sciences, Faculty of Paramedicine, Rafsanjan University of Medical Sciences, Rafsanjan, Iran

**Keywords:** Innate immunity, hepatitis B, RIP1, IPS-1

## Abstract

IPS-1 and RIP1 are the main downstream molecules of RIG1 and MDA5, as intracytoplasmic receptors, which are the main receptors involved in recognition of internal and external viral double-stranded RNA. In this project, mRNA levels of IPS-1 and RIP1 were investigated in the peripheral blood immune cells of chronic hepatitis B (CHB) patients. IPS-1 and RIP1 mRNA levels were measured in 60 CHB patients and 120 healthy subjects, using RT-qPCR technique. A significant increase in expression levels of *IPS-1* and *RIP1* was found in patients when compared to healthy individuals. There was no correlation between IPS-1 and RIP1expression levels with the serum levels of hepatitis B e-Antigen (HBeAg) and liver enzymes in patients. Based on the results, it seems that IPS-1 and RIP1 can participate in the induction of low chronic inflammation, which is a main cause of liver cirrhosis and hepatocellular carcinoma.

## Introduction

Chronic HBV-infected (CHB) patient ssuffer from mild symptoms of liver inflammation, which is a main cause of liver cirrhosis and hepatocellular carcinoma (HCC) (Chan and Jia, 2011). Accordingly, several investigations reported that the cause of cirrhosis and HCC could be CHB and the low levels of chronic immune responses to HBV (Mendy *et al.*, 2010).

The pattern recognition receptors (PRRs) identify internal, damage-associated molecular patterns (DAMPs), and external, pathogen-associated molecular patterns (PAMPs), proteins motifs (Abreu and Arditi, 2004; Sepehri *et al.*, 2016a). PRRs recognize DAMPs and PAMPs and accordingly induce expression of some pro-inflammatory molecules that either participate in the induction of appropriate immune responses against pathogens or induce pro-inflammatory-based complications such as liver cirrhosis and HCC (Zhang *et al.*, 2012; Bagheri *et al.*, 2014; Karimi-Googheri and Arababadi, 2014; Momeni *et al.*, 2014; Moreau, 2016; Sepehri *et al.*, 2016b; Sun *et al.*, 2016; Sepehri *et al.*, 2017). One of the PAMPs that is recognized by the immune system is viral dsRNA, which is produced by both DNA and RNA genomic viruses (Gerelsaikhan *et al.*, 1996; Harrison *et al.*, 2001; Park, 2004; Hu and Liu, 2017). Retinoic acid-inducible gene 1 (RIG-1) and melanoma differentiation-associated protein 5 (MDA5) are the most important PRRs that recognize viral dsRNA (Gerelsaikhan *et al.*, 1996; Harrison *et al.*, 2001; Hu and Liu, 2017) as microbial PAMPs (Hagele *et al.*, 2009; Ghosh *et al.*, 2013). MDA5 and RIG-1 have a card caspase domain (Jiang *et al.*, 2011; Triantafilou and Triantafilou, 2012) that activate signaling pathways of adaptor proteins after dsRNA recognition. IFN-β promoter stimulator-1 (IPS-1) and receptor interacting protein 1 (RIP1) are adapter molecules that activate both MDA5 and RIG-1 signaling pathways. MDA5 and RIG-1 signaling pathways are responsible for production of inflammatory cytokines, using activation of pro-inflammatory transcription factors, and consequently inhibiting virus replication (Guo *et al.*, 2009).

Accordingly, the IPS-1 and RIP-1 molecules trigger the activation of some transcription factors such as interferon regulatory factor 3 (IRF3) and IRF7, resulting in the transcription of IFN-1 and other inflammatory cytokines genes that play key roles in induction of inflammation (Szabo *et al.*, 2012). Additionally, low expression of pro-inflammatory molecules during CHB is the mechanism responsible for deterioration of CHB complications such as liver cirrhosis and HCC (Chan and Jia, 2011; Kim *et al.*, 2017; Yoo *et al.*, 2017).

Therefore, alterations in the expression of these molecules may be associated with low chronic inflammation in CHB patients. Our previous investigations revealed that mRNA levels of MDA5 and RIG-1 are decreased and increased, respectively, in CHB patients (Ebrahim *et al.*, 2015). Due to the important roles of IPS-1 and RIP1 in the intracellular signaling pathways of MDA5 and RIG-1, the alteration in MDA5 and RIG-1 may be associated with altered expression of *IPS-1* and *RIP1*. Therefore, this project aimed to investigate RIP1 and IPS-1 mRNA levels in Iranian CHB patients. The relationship between the expression of *IPS-1* and *RIP1* and serum level of liver functions markers, and hepatitis B e-Antigen (HBeAg) were determined in this project.

## Subjects and Methods

### Subjects

In this cross-sectional study, 120 healthy controls and 60 CHB patients (28 males and 32 females with age of 20-60 years old) were enrolled and referred to Razie Firroz Hospital, Kerman, Iran. The healthy controls and CHB patients were evaluated regarding IPS-1 and RIP1 expression levels and serum levels of HBeAg, HBV-DNA, and liver functional markers including aspartate aminotransferase (AST), alkaline phosphatase (ALP), alanine aminotransferase (ALT), direct bilirubin (DB), and total bilirubin (TB). Blood samples were collected from participants in 5.5-mL tubes with anticoagulant coating (to examine expression of IPS-1 and RIP1) or without anticoagulant coating (to examine HBeAg, HBV-DNA, and serum levels of liver functional markers). Based on the “Guide of Prevention and Treatment in Viral Hepatitis” (Chinese Society of Infectious Diseases and Parasitology, 2008), an internal medicine specialist diagnosed CHB based on the hepatitis B surface antigen (HBsAg) positivity for more than six months and clinical presentations. Healthy controls were matched for age and sex. Exclusion and inclusion criteria described in our previous investigations were performed on these patients (Askari *et al.*, 2016). The Ethical Committee of the Rafsanjan University of Medical Sciences certified the protocol of this study (Code: IR.RUMS.REC.1394.188), and before sample collection, a written informed consent was obtained from participants.

### Measurement of serological HBV markers

ELISA (enzyme-linked-immunosorbent-assay) kits (Behring, Marburg, Germany) were used for determination of HBsAg and HBeAg according to the manufacturer’s protocol.

### HBV-DNA extraction and qPCR assay

HBV-DNA was extracted and quantified, respectively, with commercial kits from Cinnaclon (Tehran, Iran) and Design Primer (London, UK).

### RNA extraction, cDNA synthesis, reverse transcription, and RT-qPCR

At first, for checking the expression levels of *IPS-1* and *RIP1*, total mRNA was extracted by using a commercial kit from Cinnaclon (Tehran, Iran). The quantity and quality of extracted mRNA were evaluated by spectrophotometer UV at 260-280 nm and agarose gel electrophoresis, respectively. cDNA synthesis (Parstoos Company, Tehran, Iran) and RT-qPCR (Genet Bio Company, South Korea) conditions and protocols were described completely in our previous study (Ebrahim *et al.*, 2015), except for the primer sequences which were designed using Primer3 software and are presented in Table 1. A β-actin gene was used as endogenous control for the normalization of expression levels. Changes in the expression levels of *IPS-1* and *RIP1* were reported as fold-changes (Ebrahim *et al.*, 2015).

**Table 1:** Primer sequences used in real-time PCR.

Gene		Primers
*IPS-1*	Forward	*AGCAAGAGACCAGGATCGAC*
	Reverse	GGGTATTGAAGAGATGCCAGAG
*RIP1*	Forward	AGAAAGTGTAGAAGAGGACGTG
	Reverse	AGGTACTGCCACACAATCAAG
β*-actin*	Forward	GCATGGGTCAGAAGGATTC
	Reverse	GTCCCAGTTGGTGACGAT

### Liver function tests (LFT)

For examination of the serum levels of AST, ALP, ALT, DB, and TB, Pars Azmoon commercial kits (Tehran, Iran) were used.

### Data analysis and statistical methods

The raw data for the RIP1 and IPS-1 mRNA levels were not in accordance with normal patterns, hence, the non-parametric test Mann-Whitney U test, implemented in SPSS software version 18, was used to compare CHB patients and healthy controls regarding the mRNA levels of IPS-1 and RIP1. Accordingly, Spearman’s test, as a non-parametric test, was used to evaluate the correlation between RIP1 and IPS-1 and serum levels of liver enzymes in the CHB patients. All samples were included in the statistical analysis and the significance level in the tests was set at *p*<0.05. The mRNA levels were presented using the 2^-ΔΔct^ formula, as described in our previous investigation (Ayoobi *et al.*, 2013).

## Results

### Serum levels of liver enzymes and direct/total bilirubin in CHB patients

Serum levels of ALT, ALP, AST, DB, and TB were evaluated in these patients in our previous investigation, so the data was presented in a previous article (Askari *et al.*, 2016).

### HBV serum markers

All patients were positive for HBsAg, while HBeAg was positive in only 4 (6.66%) CHB patients.

Because viral load has significant effects on the expression of immune-related molecules (Michalak *et al.*, 2000), HBV-DNA viral load was evaluated in this study.

### IPS-1 and RIP1 mRNA levels in patients and controls

**Figure 1:**
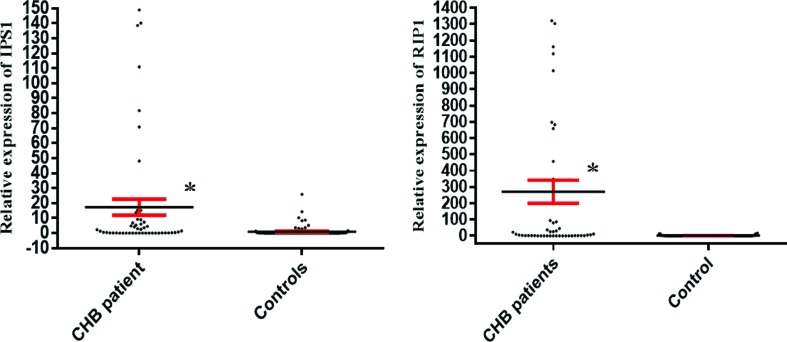
*IPS-1* and *RIP1* gene expression in patients with chronic hepatitis B and controls. IPS-1 and RIP1 mRNA levels were significantly increased in patients when compared to healthy controls. Data are presented as mean ± standard errors. The results regarding the expression levels of IPS-1 and RIP1 are reported as fold-changes (Ebrahim *et al.*, 2015). *p-value < 0.001

The IPS-1 mRNA level in CHB patients was 1.9279 (range 0.1429-11.2525) and in healthy subjects it was 0.0672 (0.0185-0.1438) (Figure 1). The difference between the two groups was statistically significant (*p*<0.001).

RIP1 mRNA levels in CHB patients were significantly (*p*<0.001) increased (16.7373; range (0.8017-677.2051) in comparison to healthy subjects (2.7406; range 0.2499-9.1961). Figure 1 illustrates IPS-1 and RIP1 mRNA levels in CHB and healthy controls.

### Expression of IPS-1 and RIP1 genes in male and female patients

**Figure 2:**
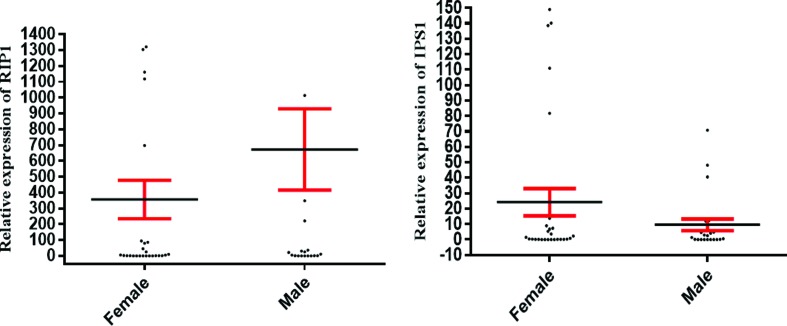
*RIP1* and *IPS-1* gene expression in male and female patients with chronic hepatitis B. The figure shows that mRNA levels of IPS-1 and RIP1 did not differ between male and female patients with chronic hepatitis B. Data are presented as mean ± standard errors. The results regarding the expression levels of IPS-1 and RIP1 are reported as fold-changes (Ebrahim *et al.*, 2015).

Mann-Whitney’s test showed that there were no significant differences in the expression of *RIP1* (*p*= 0.272) and *IPS-1* (*p*= 0.665) in both male (28 cases) and female (32 cases) patients (Figure 2).

### IPS-1 and RIP1 mRNA levels in the HBeAg-positive and negative CHB patients

**Figure 3:**
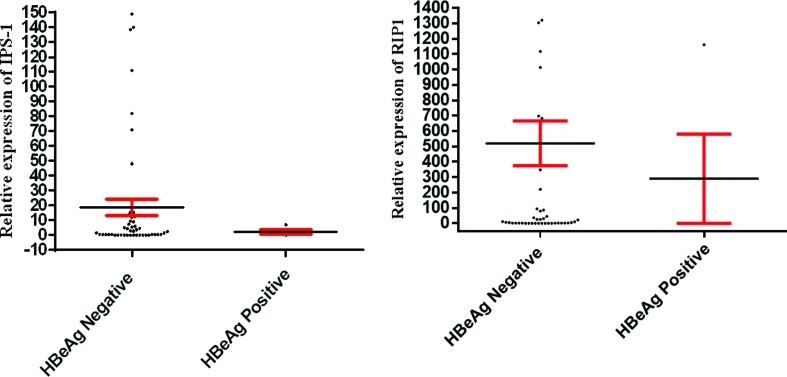
*IPS-1* and *RIP1* gene expression in HbeAg-positive and negative patients with chronic hepatitis B. The figure shows that HBeAg positive and negative patients did not show differences in their IPS-1 and RIP1 mRNA levels. Data are presented as mean ± standard errors. The results regarding the expression levels of *IPS-1* and *RIP1* are reported as fold-changes (Ebrahim *et al.*, 2015).

The median expression of *IPS-1* and *RIP1* in HBeAg positive CHB patients was 22.00 and 15.75, respectively, while these values in HBeAg-negative patients were 26.88 and 27.47. Statistical analysis showed that the differences were not significant for the expression of *IPS-1* (*p*= 0.562) and *RIP1* (*p*= 0.369) (Figure 3).

### Relationship between IPS-1 and RIP1 gene expressions with liver functional markers in CHB patients

Spearman’s test was used to determine the correlation between *IPS-*1 and *RIP1* gene expression with serum levels of liver enzymes. Based on the data presented in Table 2, there was no significant correlation between expression of the two genes and the liver functional markers.

**Table 2:** Associations of IPS-1 and RIP1 genes with serum levels of liver enzymes.

			Viral copy number	IPS1	RIP1	DB	TB	ALT	ALK	AST
Spearman’s rho	Viral copy	Correlation coefficient	1.000	0.142	0.197	0.208	0.163	0.093	0.126	0.132
	number	*p*-value	-	0.348	0.190	0.139	0.249	0.513	0.375	0.351
	IPS-1	Correlation coefficient	0.142	1.000	0.757^*^	0.094	-0.071	0.125	0.099	0.275^**^
		*p*-value	0.348	-	0.000	0.507	0.616	0.377	0.485	0.049
	RIP1	Correlation coefficient	0.197	0.757^*^	1.000	0.256	0.113	0.087	0.173	0.212
		*p*-value	0.190	0.000	-	0.067	0.423	0.542	0.221	0.131

Spearman’s test revealed that there were significant, yet poor positive correlations between IPS-1 and RIP1 (*) and between IPS-1 and AST (**), respectively.

## Discussion

The results showed that mRNA levels of IPS-1 and RIP1 significantly increased in the CHB patients. Because CHB patients suffered from chronic inflammation, and this is a reason for induction of liver cirrhosis and hepatocellular carcinoma (HCC), the most important CHB complications, it may be hypothesized that the up-regulation of *IPS-1* and *RIP1* expression is a crucial mechanism to induce or stimulate chronic inflammation, and consequently liver cirrhosis and HCC. Interestingly, our previous investigation on the same CHB patients revealed that the mRNA levels of RIG-1, the upstream molecule of IPS-1 and RIP1, was significantly increased, while MDA5 mRNA levels were decreased (Ebrahim *et al.*, 2015). Because RIG-1 and MDA5 are the innate immune receptors that recognize microbial PAMPs and then activate downstream molecules, including IPS-1 and RIP1, which consequently result in activation of pro-inflammatory transcription factors such as IRF3 and IRF7 (Reikine *et al.*, 2014; Li *et al.*, 2018), it seems that RIG-1/MDA5 and its downstream molecules can be considered as unknown parts of the CHB-related liver cirrhosis and HCC puzzle. Interestingly, there is evidence in favor of this hypothesis. For example, Zhao *et al.* (2012) used monocyte-derived dendritic cells (moDCs) that had been derived from CHB patients to evaluate expression of *RIG-1* and *IPS-1*. They reported that, although expression of *RIG-1* decreased in the moDCs, stimulation of the cells with vesicular stomatitis virus (VSV) in *in vitro* condition led to an up-regulation of both *RIG-1* and *IPS-1* after 8 and 16 hours, respectively. However, the expression of *RIG-1* was decreased after stimulation of the cells for 16 hours. As IPS-1 levels were higher at 16 hours after stimulation and did not decrease, it may be hypothesized that IPS-1 is induced by other unknown pathways. Moreover, it also can be concluded that IPS-1 participates in the induction of inflammation more than RIG-1, which needs to be explored by additional studies. Ye *et al.* (2012) also showed that RIP1 plays an important role in the induction of cirrhosis: RIP1 releases cytochrome C from mitochondria and, through TNF-α, causes ROS and mitochondrial dysfunction, resulting in necrosis, which is seen in the liver of the patients who suffer from liver cirrhosis. While the results revealed a certain but rather minor relationship between IPS-1 and AST, there was no a significant relation ship between mRNA levels of IPS-1 and RIP1 and liver function markers (Table 2). Nonetheless, a positive relationship between ALT and RIG- 1/IPS-1 has been reported (Zhao *et al.*, 2012).

As all of the patients who participated in the current investigation had normal ranges of liver function markers, there was no relationship between IPS-1 and RIP1 mRNA levels and serum levels of liver function markers were not significant. However, previous investigations revealed that AST is a critical marker of liver inflammation, which may be associated with liver cirrhosis and HCC (Wang *et al.*, 2018). Due to the positive correlation between AST and IPS-1, it may be hypothesized that IPS-1 is an inducer of liver inflammation. Accordingly, following the patients in a cohort investigation could be useful to clarify the roles played by IPS-1 and RIP1 and their up/down-stream molecules in the pathogenesis of chronic hepatitis.

HBeAg was not associated with the expression of *IPS-1* and *RIP1*. Our previous investigations on RIG-1 and MDA5 had similar results and revealed that there was no relationship between the expression of HBeAg by HBV and expression of MDA5 and RIG-1 by host immune cells (Ebrahim *et al.*, 2015). Interestingly, our previous investigations on other innate immunity molecules showed that there was no association between the expression of innate immunity-related molecules such as toll-like receptor 9 (TLR9) and its downstream molecules with the expression of HBeAg (Momeni *et al.*, 2014; Askari *et al.*, 2016). Thus, it may be hypothesized that HBeAg does not alter the expression of innate immunity-related molecules in Iranian CHB patients.

Additionally, there was no significant relationship between mRNA levels of IPS-1/RIP1 and gender for the CHB patients. Our previous investigations also had the same results and revealed that mRNA levels of MDA5, RIG-1, TLR9, myeloid differentiation primary response gene 88 (MYD88), TIR-domain-containing adapter-inducing interferon-β (TRIF), Interleukin-1 receptor-associated kinase 1 (IRAK1), IRAK4, tumor necrosis factor receptor-associated factor 3 (TRAF3), TRAF6, nuclear factor B (NF-κB), and interferon regulatory factor 7 (IRF7) were not associated with the participant’s gender (Ayoobi *et al.*, 2013; Sajadi *et al.*, 2013; Momeni *et al.*, 2014). Although several investigations proved that gender is a factor that can alter the expression of immune-related molecules (Oertelt-Prigione, 2012; Klein and Flanagan, 2016; Ruggieri *et al.*, 2016), our investigations were unable to show such differences in male and female CHB patients. The result may be related to the infectivity to HBV, different genetic status, and environmental factors that need to be explored by further investigations. Thus, it seems that gender is not associated with the expression of innate immunity molecules in Iranian CHB patients. Additionally, because high variation for immune-related molecules is common (Ayoobi *et al.*, 2013; Sajadi *et al.*, 2013; Momeni *et al.*, 2014), increasing the sample size may reveal significant differences between groups (based on gender and HBeAg positivity) for these molecules of interest.
